# Anatomical Variations in the Pectoralis Minor Muscle Origin and Insertion: A Systematic Review

**DOI:** 10.7759/cureus.46329

**Published:** 2023-10-01

**Authors:** Yasser AlHarbi

**Affiliations:** 1 Department of Basic Medical Sciences, College of Medicine, King Saud Bin Abdul Aziz University for Health Sciences (KSAU-HS), Jeddah, SAU

**Keywords:** shoulder joint capsule, glenohumeral joint capsule, scapula, coracoid process, pectoralis minor muscle

## Abstract

The pathology of the shoulder is among the most widespread medical presentations and may be a result of existing anatomical variations. Therefore, the knowledge of the variations is vital for physicians and clinicians, tasked with treating patients presenting similar complaints to minimize misdiagnosis and prevent iatrogenic injuries. Therefore, the main objective of the present systematic review the variations in pectoralis minor muscle origin and insertion/attachment point. The study also seeks to better inform physicians and clinicians of the task of treating patients with various pathology problems and to ascertain that, upon identification, the pectoralis minor muscle variants are aptly appreciated. The search method used in this systematic review entails the use of the Preferred Reporting Items for Systematic Reviews and Meta-Analyses (PRISMA) protocol, and the searching of several online databases, for studies focusing on variations in pectoralis minor muscles. The author reviewer evaluated the studies for eligibility, and the selection criteria for the studies used are described below. This systematic review has disclosed that, in some individuals, the pectoralis minor muscles have their origins in the second, third, and fourth ribs, even as others have their origin in the third and fourth ribs. Still, the systematic review has disclosed that, in certain individuals, the insertion of the pectoralis minor muscle occurs at the supraspinatus tendon, even as there are anomalies in the pectoralis minor insertion points linked to subacromial impingement, possible compression of the brachial plexus anteromedial and the axillary artery, and the subcoracoid impingement.

## Introduction and background

The pectoralis minor refers to the thinner and rectangular-shaped chest muscle that is located deep and posterior to the pectoralis major muscle. The pectoralis minor is mainly attached inferiorly to the third to the fifth ribs close to the costal cartilages, in addition to being superiorly attached to the scapula’s coracoid process and forming part of the axilla’s anterior wall. The pectoralis minor anatomically offers the landmark to the much deeper structures that include the medial pectoral nerves, the axillary arteries, and the brachial plexus cords. Several anatomy studies and literature have reported variations with regard to the pectoralis minor muscle attachment points [1-3].

Le Double, a French anatomist developed and wrote the treaty on anatomical variations, especially muscle variations, and offered an in-depth description of three kinds of muscle variations related to the proximal pectoralis minor muscle attachments in 1897 [4]. In the study, Le Double categorized the insertional variants of the PMi muscles into three key types, with Type 1 involving the deeper part of the PMi muscle insertion occurring at the coracoid process in the normal fashion, even as the superficial part of the PMi crosses over it to an increasingly proximal structure. Type 2 variation entails the insertion of a larger proportion of the muscle fiber occurring at the coracoid process, with a few muscle fibers crossing over the coracoid process. Type 3 variation involves the PMi muscle crossing over the coracoid process as a single unit devoid of inserting at all and is detached from the coracoid process via a bursa. Le Double’s study disclosed that the Type 1 PMi variant was the most common, while Type 2 was uncommon, and Type 3 was rare [4]; nevertheless, more recent literature has disclosed that the Type 2 PMi variant was less uncommon than initially believed [5]. Still, in recent times, a limited number of publications have been able to report such pectoralis minor variations in different anatomic studies. In this regard, several authors have linked the pectoralis minor attachments and insertion point variations to the development of rotator cuff syndrome, which occurs during surgical procedures [6,7]. For instance, ultrasound-based research disclosed a 9.57% frequency with regard to the detection of variations in pectoralis minor insertions [8,9]. The correlations between the coracohumeral ligament and the pectoralis minor end have been highlighted by several researchers. Such variations that are, in clinical practice, normally discovered in the course of rotator cuff syndrome surgery, should be considered during the diagnosis of mechanical impingement [10,11].

Moreover, the variations in the pectoral region’s accessory muscles, which intervene between the pectoralis minor and pectoralis major, have been noted in studies using human cadaveric specimens. The variations include pectoralis intermedius, pectoralis minimus, pectoralis quartus, and pectoralis tertius. Thus, pectoralis minimus has its origin in the first and second ribs with the insertion occurring on the scapula’s coracoid process [12]. Consequently, the pectoralis intermedius has its origin in the third and fourth ribs and between the pectoralis minor and pectoralis major muscles, with the insertion occurring on the scapula’s coracoid process [2,11,13]. On the contrary, pectoralis Tertius has its origin in the lower ribs with insertion occurring on the scapula’s coracoid process and the humerus, while pectoralis quartus has its origin in the lower ribs and on the rectus sheath and pectoralis major muscle’s lateral border with the insertion occurring in the bicipital groove and the upper arm fascia [2,3]. In this regard, the other objective of the present study is to identify the correlations between the variations in pectoralis minor muscles and the variations in the pectoral region’s accessory muscles (intermediate pectoralis muscle (IPM)). Further, in this regard, the present study has reported the existence of the pectoral region’s accessory muscles with the variations in the pectoralis minor muscles’ insertion points.

The observed anatomical variations with regard to the pectoralis minor muscle might present considerable implications during surgical procedures like the modified radical mastectomy procedure. Moreover, the pectoralis minor muscles have often been utilized as the muscle flapping with respect to the correction and reconstruction of the anterior shoulder and the facial palsy [12,14,15]. The present study has, therefore, reported the variations in the attachment of the pectoralis minor that have to be taken into account during the performance of surgical procedures on the axillary region of the human body and the chest wall. The study has also disclosed that the major pectoralis minor muscle variations in humans are found at the insertion points that include the humerus’ greater tubercle, the supraspinatus tendon, the coracoacromial ligament, and the glenohumeral capsule.

## Review

Materials and methods

The present systematic review was performed as per the PRISMA guidelines for reporting systematic reviews and meta-analyses of studies on human anatomy.

Search Strategy

The literature search conducted lasted until December 2022 and was mainly performed through various electronic databases that included EMBASE, Medline, PubMed, Scopus, Science Direct, Cochrane Library, and Web of Science. The search was conducted with the objective of identifying anatomy studies eligible for inclusion in this systematic review. Among the terminologies used in conducting the literature search included pectoralis minor muscles, anomalous variations, coracoacromial ligament, coracoid process, and glenohumeral capsule. For each relevant and eligible study identified, a further manual search was conducted on the reference list for additional relevant studies for inclusion in the systematic review.

Study Selection Criteria

For inclusion in the systematic review, studies were considered eligible if they: reported extractable data or information regarding the divergences in pectoralis minor muscle origins and insertion points, and presented aptly and accurately defined descriptions of the pectoralis minor muscle variations. The criterion aimed at ascertaining that only studies with appropriate and verified knowledge regarding variations in pectoralis minor muscles are used in the systematic review. Further, the criterion for inclusion of the studies entailed ensuring that only studies published in English were used in the systematic review. The basis of the criterion regards the need to ensure that only the most appropriate and comprehensible information is included in the systematic review. Lastly, the criteria entailed the inclusion of studies available as full-texts, and excluding those that only provide previews and abstracts. The authors have acknowledged that most research article previews and abstracts have been exceedingly simplified to aspects of the research questions, while each study content tackled variable factors and approaches to responding to the research questions, which are essential for the current study. For this systematic review, only peer-reviewed journal articles were considered, and books, editorials, web articles, newspaper commentaries/articles, and book reviews were excluded. The basis of this criteria was the need to ensure the use of empirical studies that were more comprehensive in their approach to the concepts sought after by the systematic review.

Data Extractions

For every study, the two authors (who doubled up as reviewers), working independently, carried out extensive database research, extracted the necessary data, and subsequently entered it into Excel datasheets. In case of discrepancies, a third reviewer was requested to review the article and assist in determining its suitability and eligibility. Further, from each article, the following data was collected and summarized; study title; authors’ names; publishing year; the overall number of participants/cases; the inclusion/exclusion criteria used; the number of participants/cases with different types of pectoralis minor muscle variations (including the pectoralis minor muscle origin and the insertion point).

Assessment of the Studies’ Quality

To conduct the quality assessment of the identified studies, the Quality in Prognostic Studies Tool16’s four scales, including reporting and statistical analysis, study participation, outcome measurement, prognostic factor measurement, and confounding. Further, for each scale, a subscale was developed to enable the authors to grade the studies as low quality (0), intermediate quality [1], and high quality [2]. The quality assessment approach has been widely used by authors who have conducted systematic reviews and meta-analyses of variations of arteries and different body muscles [16].

Statistical Analysis

All statistical analyses were conducted using the random effects model. Moreover, the I2 statistic and Chi2 tests were employed in the evaluation of heterogeneity between the studies. Also, regarding the Chi-2 test, the p-value of < 0.05 was regarded as indicating statistically significant heterogeneity between studies [17-43].

Results

Search Synthesis

The initial database search yielded a total of 240 articles, and after the deletion of irrelevant and duplicate studies, a total of 188 were obtained for further screening and review. Through the analysis of the selected studies’ references, I discovered another 22 possibly pertinent articles that I obtained and added. From the 210 articles, after the review, only 49 were included in the systematic review and meta-analysis of anatomical variations in pectoralis minor muscles origin and insertion points, out of which all had data regarding the variations in pectoralis minor muscles.

Evaluation of Articles Quality and the Risk of Bias

**Figure 1:**
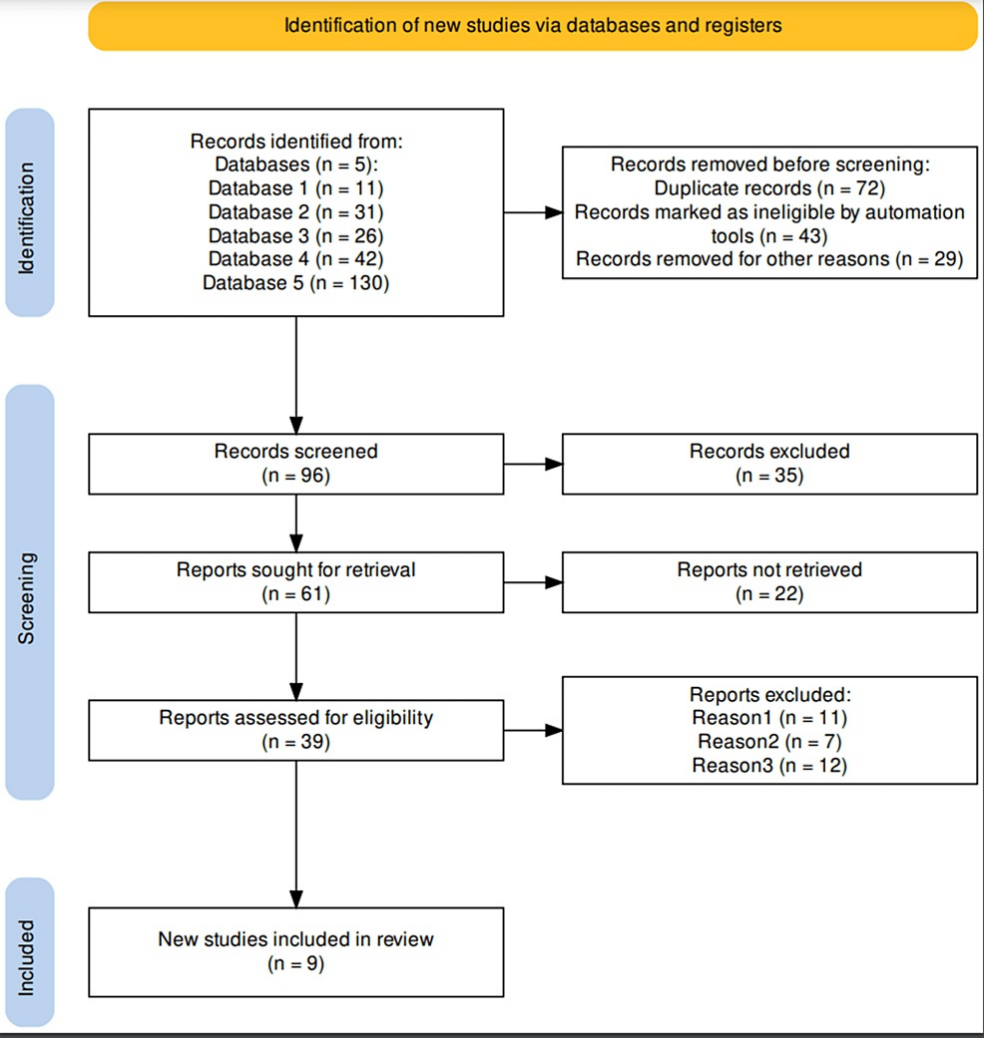
PRISMA flow diagram PRISMA flow diagram that indicates that study selection and inclusion criteria for the studies reviewed in this systematic review

Based on the established inclusion and exclusion criteria, a total of 49 studies were obtained, out of which a further screening for high quality (6 to 8 points rated) articles disclosed 28 articles, 19 medium quality articles rated between 3 and 6, and two low-quality articles rated between 0 and 2 points. Figure 1 is the PRISMA flow diagram indicating the literature search and selection process.

Table 1 indicates the various studies selected and reviewed in this systematic review.

**Table 1:** List of studies included in this systematic review. Studies that have been selected and reviewed in this systematic review, including information such as author name, study title, study objective, study design, publication year, and sample size.

Author	Study Title	Objectives	Research Design	Sample Size	Publication year	Journal
Burley et al. [10]	The clinical anatomy of variations of the pectoralis minor.	To review the variant anatomy of the pectoralis minor muscle with the objective of better informing clinicians who might treat patients with shoulder pathology so that if identified, variants of the pectoralis minor muscle are better appreciated.	Cadaveric research	115 cadavers	2021	Surgical and Radiologic Anatomy
Asghar et al. [12]	The Anomalous Insertion of Pectoralis Minor (Le Double Type III): A case report.	To review the curious case of the anomalous pectoralis minor muscle insertion at greater tuberosity via rotator interval.	Case Study (Cadaveric research)	One 60 year old male cadaver	2021	Anatomical Sciences Journal
Goldman et al. [15]	Unilateral ectopic insertion of the pectoralis minor: Clinical and functional significance.	To review the unilateral ectopic insertion of the pectoralis minor muscles, including the aponeurosis extension from the tendon of the pectoralis minor muscles and attaching to the coracoid process.	Case study (Cadaveric research)	One 70 year old female cadaver	2016	Morphologie
Asghar et al. [16]	The prevalence and distribution of the variants of pectoralis minor in cadaveric studies: A systematic review and meta-analysis of 32 observational studies.	To provide in-depth insights to the surgeons with regard to the variants of PMi in terms of costal attachment, insertion, nerve supply, and arterial supply.	Systematic Review and Meta-Analysis		2021	National Journal of Clinical Anatomy
Sanchez et al. [29]	Anatomic relationship of the pectoralis major and minor muscles: a cadaveric study.	To identify the anatomic relationship of the costal origins of the pectoralis major and minor muscles.	Cadaveric research	102 cadavers	2014	Aesthetic Surgery Journal
Lim et al. [32]	Pectoralis minor tendon in the rotator interval: arthroscopic, magnetic resonance imaging findings, and clinical significance.	To evaluate the prevalence of tendinous insertion of the pectoralis minor by arthroscopy and diagnostic performances of MRI and suggests its clinical implication in rotator cuff repair.	Observational research	99 patients	2015	Journal of Shoulder and Elbow Surgery
Schwarz, Hirtler [33]	Ectopic tendons of the pectoralis minor muscle as cause for shoulder pain and motion inhibition—explaining clinically important variabilities through phylogenesis.	To evaluate the clinical consequences of ectopic tendons of the pectoralis minor muscle (PMM) for shoulder pain and range-of-motion limitation.	Cadaveric research	45 shoulders	2019	PLoS One
Keet, Louw [35]	Superficial location of the brachial plexus and axillary artery in relation to pectoralis minor: a case report.	To review the variants of the pectoralis minor and major muscles within the infraclavicular fossa.	Cadaveric research	170 dissections	2014	Korean Journal of Radiology
Lee et al. [38]	Ectopic insertion of the pectoralis minor tendon: inter-reader agreement and findings in the rotator interval on MRI.	To evaluate magnetic resonance imaging (MRI) observer variability to detect ectopic insertion of the pectoralis minor tendon (EIPMT) and to investigate changes in the rotator interval in patients with EIPMT using MRI.	Mixed methods research	507 shoulder MRIs	2014	Korean Journal of Radiology,

Discussion

The pectoralis minor muscle variants recorded in existing literature have been linked to the muscles’ distinctive insertions on structures other than the scapula’s coracoid process. Such ectopic insertions include coracoacromial ligaments, clavicle, supraspinatus tendon, and humeral tubercles [2]. A number of the muscle variations that have been reported in existent literature include those that are mainly discovered in the course of regular cadaveric dissections and are of immense importance to physicians, anatomists, and biologists (developmental) [8,18]. Nonetheless, the recognition and comprehension of the pectoralis minor muscle are normal and variant anatomy is of vital significance to physicians and healthcare professionals during diagnosis, surgical treatment, and imaging of the shoulder and surrounding regions.

Variations in Pectoralis Minor Muscle Origin

The approved “typical” origin of the PMi muscle is the third to the fifth ribs [5,19,20]. Nevertheless, the origin of PMi at the second to the fourth ribs is comparatively common, even as evidence regarding gender differences in relation to the locations where the PMi develops the inferior attachments exist [21,22]. For instance, in a published cadaveric study by Hojo, Nakashima, and Tsuruno, it has been noted that, in males, the most common PMi muscle origin was the third to the fifth ribs while in females the origin was in the second to the fourth ribs [21]. Still, Hojo et al. disclosed that, in Japanese males, the PMi origin was the second to the fifth ribs, while in Japanese women the PMi origin was the second to the fourth ribs [21]. Regardless of whether such distributions are attributable to the anticipated divergences between the genders or the high rates of PMi variants in females remains arguable [8]. Also, anomalous PMi attachments from the first to the seventh ribs have been observed and documented [23]. Documentations of the variations with regard to the inferior PMi origins are limited in extant literature. For instance, a case regarding the right PMi originating from only the fifth rib, devoid of any other anatomical variations has been reported by Turan-Ozdemir and Cankur [20]. Occasional origin of the PMi at the sixth rib has also been reported. Such variations in the origins of the PMi are always clinically silent.

The superior origin variations have been of immense interest and have been the subject of many studies, including research by Wood who reported an instance in which the PMi muscle’s superior origin was distinct from other parts of the muscle, and extended from the initial intercostal aponeurosis to the clavicle and costocoracoid membrane [24]. A similar study reported fibers originating from the clavicle and whose attachment occurred at the second rib and approaching the sternoclavicularis muscle formation [25]. Also, a dissection study conducted by Fokin et al. reported the right PMi that originated from the second to the fifth ribs and was inserted at the shoulder joint capsule and the coracoid process, even as the left PMi muscles were totally absent and had been replaced with a thicker fascia [26]. In a study conducted by Lee et al, the researchers disclosed that in two of the 32 samples, the origin of the PMi was at the first rib, and in 26 of the 32 samples, the origin of PMi was at the second rib [5]. Surprisingly, in their respective studies, Mori [27] and Motwani et al. [28] observed that, given that apes usually have a minimum of four and a maximum of seven rib attachments for PMi, while the number of ribs has been reduced in humans. A cadaveric dissection study conducted by Sanchez et al. involved 202 PMi muscles and focused on the distance between the muscles’ costal attachments [29]. Thus, 24% of the specimens used in the study were observed to have a distance of below 1 cm while 41% of the specimen had a distance of 1 to 3 cm, and the remaining 35% had a distance above 3 cm.

Variation to Pectoralis Minor Muscles Attachment

The anomalous pectoralis minor muscles origin and insertion patterns have been studied through the use of different methodologies that include MR arthroscopy, MRI, ultrasound, and dissection, even as dissection has been observed to be more effective compared to the various non-invasive methodologies. Asghar et al. initially reported the pectoralis minor muscle insertion into the shoulder joint capsule [16]. The anomalous pectoralis minor muscle tendon has also been reported to make a groove at the coracoid process, besides blending into the fibrous capsule together with the supraspinatus [16]. Studies have additionally reported instances where distinct pectoralis minor muscle’s aponeurosis has crossed the coracoid process’ root and subsequently wounded outward and downward and inserted into the shoulder capsule [24-29]. Initial observations regarding the anomalous variations of the pectoralis minor muscles origin and insertion points have been reported by Le Double and Testut, who reported on the different types of PMi insertions [4,30].

A study conducted by Lee et al. using MR arthroscopic assessment of 335 shoulders disclosed five types III insertions, with one being muscular and four being tendinous while crossing over the coracoid process [5]. Nevertheless, of the 335 shoulders, 74 cases were classified as type III, indicating a high prevalence. In instances where the variation in the insertion or attachment is classified as a Le Double type III, it is highly prone to be concomitant with a lack of the coracohumeral ligament [3,4,31]. In their study, Tubbs et al. reported an instance in which the origin of the pectoralis minor muscle was at the normal third to the fifth costochondral junctions, before crossing over the coracoid process devoid of direct attachment or insertion into the capsule of the fibrous joint [31]. On the contrary, examination of the right shoulder disclosed no abnormality with regard to the musculoskeletal. Still, in their study on the classification of unusual insertion of the pectoralis minor muscle, in which 74 cadavers were dissected and studied, Lee et al. observed that, in 23% of the cadaveric samples, there were anomalous insertion patterns [5]. Thus, the study disclosed that, in instances where part of the pectoralis minor muscle was observed to run over the coracoid process, the glenohumeral joint capsule was the most common site of attachment. Further, the study by Lee et al. also confirmed the pectoralis minor muscles' attachment to the clavicles, even as the pectoralis minor muscles’ coastal attachments were frequently noted to extend from the second to the fourth rib [5]. Further, the study by Lee et al. disclosed bilateral symmetric variations of the pectoralis minor muscle insertion points, which include the tendon crossing over the coracoid process’ superior surface before fusing with supraspinatus muscle’s tendon and before insertion onto the humerus head’s major tubercle [5].

The above observations are in concurrence with Le Double’s description of the three notable types of anomalous pectoralis minor muscle insertions. Moreover, in a study conducted by Lim et al., the pectoralis minor muscle’s ectopic tendon was found during exploratory shoulder surgery in an individual suffering from shoulder pains during movement, with the cause being noted as conventional and radiological semiotic examination [32]. Still, Schwarz and Hirtler have documented insertions and attachments that are considerably more distal compared to the glenohumeral joint capsule at the humerus’ greater tubercle [33]. In such instances, the insertion of the pectoralis minor muscle occurs via the supraspinatus tendon. Still, an earlier study conducted by Low and Tan in 2010 documented a prevalence rate of such variations of nearly 0.1%, even as Lee et al. documented a case of PMi insertion at the humerus’ greater tubercle in 1 out of the 74 shoulders [34]. Also, Keet and Louw, and Lee et al. noted anomalous insertion of the PMi in nearly 23% of the total number of dissections they conducted [35,36]. The supraspinatus tendon insertion is likely to result in the imposition of undue tension on the rotator cuff while negatively affecting the repair of the rotator cuff.

The anomalous insertion of PMi has been observed to vary in prevalence on the basis of gender, equipment utilized, the side of the body involved, and the absence and existence of symptoms [8,35]. For instance, a study conducted in the United States disclosed anomalous PMi insertion in 9.58% of the dissected and evaluated shoulders in 40 patients, including 22 unilateral patients and 18 bilateral patients [8]. A statistically significant prevalence of anomalous insertions of PMi was observed on the left side shoulder at 12.2% (in 37 out of 303 patients) in comparison to the right-side shoulder at 6.9% (in 21 out of 303 shoulders), as well as for females at 12.2% (in 42 out of 392 patients) in comparison to the males at 4.7% (in 10 out of 214 patients). The study did not disclose any considerable correlation between the anomaly's existence and the various clinical symptoms [8]. In one of the recent studies carried out in relation to PMi muscle variations in humans and different apes, Diogo et al. discovered that in orangutans, gorillas, and hylobatids, the insertion of the PMi occurred on the scapula’s coracoid process and that in Pan troglodytes, the most common insertion point was closer to the humerus and also at the glenohumeral capsule [12]: nonetheless, certain researches have documented partial or complete PMi insertion at the scapula’s coracoid process [36]. Further, in a comparable study conducted by Potau et al., it was reported that all Pan Troglodytes had similar, comparatively anomalous patterns of PMi insertions, in which the PMi muscle tendons crossed over the coracoid process before final insertion at the supraspinatus tendon [37]. Further, the study disclosed that in the Pan paniscus specimen studied, the PMi muscle insertion occurred at the scapula’s coracoid process, which corroborates the findings of initial studies that noted that, similar to the bonobos, in humans, the most common PMi muscle insertion point was the scapula’s coracoid process [37]. Nevertheless, other notable insertion points have been noted with regard to human PMi muscles, including the humerus’ greater tubercle, the coracoacromial ligament, and the glenohumeral capsule [15,29,38]. Potau et al. reported that, in two human cadaver specimens dissected, the insertion of the PMi occurred at the glenohumeral capsule after crossing over the coracoid process [37]. Additionally, in humans, the PMis has been noted to insert at the supraspinatus tendons, and this is comparable to the findings made with regard to Pan Troglodytes [39,40]. Still, a number of studies have disclosed the existence of numerous anomalies in the insertion of PMi in humans associated with subacromial impingement, shoulder stiffness and pain, and limited glenohumeral joint lateral rotation [7,41,42].

Infrequently, the PMi muscles have been observed to insert on the humeral tuberosity or the glenohumeral joint capsule. In this regard, Rouvière and Delmas noted that, from the phylogenetic point of view, the coracohumeral ligament can be observed to be the result of the post-coracoid/distal PMi muscle fibers [43]. In their study conducted in 1986, Weinstabl et al. showed the continuity that existed between the PMi and the coracoglenoidal ligament, which had the muscle fibers crossing over the coracoid process in approximately 16% of the assessed 126 anatomical samples [44]. Further, a number of studies have noted the continuity between the PMi tendon fibers and the coracoglenoidal ligaments in evaluated 27 out of the 34 samples [45-47]. Still, Turgut et al. noted an instance of anomalous bilateral insertion of the PMi, which entailed the lying of the variant muscle under the pectoralis major, in addition to being medial to the PMi [47]. The tendon’s thicker part, comparable to the anomalous PMi tendon insertion, crossed over the coracoid process and was attached to the shoulder joint’s articular capsule, even as, on the left side, the thinner part was attached to the clavicle’s lateral third [3,47].

**Table 2:** A summary of the different anatomical variations of the pectoralis minor muscles.

Variation Type	Description
Variations in Pectoralis Minor Muscle Origin	The variations are depicted in different genders with PMi muscle originate between the 3^rd^ and the 5th ribs in males, and between the 2^nd^ and the 4^th^ ribs in females. In Japanese, the PMi muscle mostly originate from the 2^nd^ to the 5^th^ ribs in males, and the 2^nd^ to the 4^th^ ribs in females. Higher rates of PMi muscle origin variants have been recorded in females than males.
Variations in PMi Muscle attachment	The variation in PMi muscle attachment is mainly marked by the anomalous PMi attachments from the 1st to the 7th ribs.
Variations regarding the inferior PMi origins	The variation type is marked by the right PMi originating from only the 5th rib, devoid of any other anatomical variations. Infrequent origin of the PMi at the 6th rib has also been reported.
Superior origin variations	The variation type involves: The PMi muscle’s superior origin that is distinct from other parts of the muscle, and extending from the initial intercostal aponeurosis to the clavicle and costocoracoid membrane. The PMi muscle fibers originating from the clavicle with the attachment occurring at the 2nd rib, and approaching the sternoclavicularis muscle formation. The right PMi muscle originating from the 2nd to the 5th ribs and attaching at the shoulder joint capsule and the coracoid process, with the left PMi muscles being non-existent and replaced with a thicker fascia.
Variation to PMi Muscles attachment.	The variation type is mainly marked by: The pectoralis minor muscle attachment at the shoulder joint capsule. Anomalous pectoralis minor muscle tendons making grooves at the coracoid process, in addition to blending into the fibrous capsule together with the supraspinatus. Distinct pectoralis minor muscle’s aponeurosis crossing over the coracoid process’ root and wounding both outwards and downwards, and attaching to the shoulder capsule. The PMi muscle origin is at the normal 3^rd^ to the 5^th^ costochondral junctions, before crossing over the coracoid process without direct attachment to the capsule of the fibrous joint. The PMi muscles attachment to the humeral tuberosity or the glenohumeral joint capsule. A part of the PMi muscle running over the coracoid process and attaching the glenohumeral joint capsule. The PMi muscles attaching to the clavicles, even as the PMi muscles’ coastal attachments frequently extending from the 2^nd^ to the 4^th^ rib. The Bilateral symmetric variations of the PMi muscle attachment points, including the tendon crossing over the coracoid process’ superior surface before fusing with supraspinatus muscle’s tendon and before attaching to the humerus head’s major tubercle.
PMi muscle attachments that are more distal than the glenohumeral joint capsule at the humerus’ greater tubercle.	The variation is marked by: The attachment of the PMi muscle occuring via supraspinatus tendon. The PMi attachment occuring at the humerus’ greater tubercle in the shoulder.

Implications of the Variations of the PMi Muscles to Clinical Practice

Normally, anatomical variations do not have any impact on the functioning of organs or the body. However, in clinical practice, anatomical variations are known to play vital roles with regard to the diagnosis and treatment or therapeutic procedures, as they might require additional attention and specific arrangements [20,31]. Close relations have been reported between anatomical variations and race, geographical region, exposure to radiation, and chemical, and environmental adaptations. Therefore, the identification of such variations is prone to reflect positively on clinical care outcomes through the reduction of malpractices and confusion. The PMi muscles have shown variable anatomy that has to be considered in clinical practice, given that the PMi is an important anatomical landmark found on the chest walls and provides access to several associated structures in the axial and clavipectoral regions. Nevertheless, the PMi muscles have indicated variations in the origin and attachment/insertion sites, length, innervation, and vasculature [20,24-27].

Still, the anatomical variations of the PMi muscles are prone to influence the predisposition of individuals to diseases, clinical assessment, symptomatology, investigation, and management of patients including operative surgeries [36-40]. The accurate knowledge of the variations in the PMi muscles is important in the enhancement of both diagnostic and interventional performance, particularly against modern imaging techniques backgrounds, including MRI, echocardiography, CT scan, laparoscopic and open surgery, and endoscopy [23,42]. Several reports have attributed a considerable percentage of clinical practice to a lack of knowledge of anatomical variations [42]. Thus, the understanding of the variations in PMi muscles is important to surgeons and other physicians when conducting operations on the chest and shoulder regions, as well as radiologists tasked with the interpretation of CT scans and radiographic films. Through understanding the anatomical variations of the PMi muscles, the operative inadvertent damages to the chest and shoulder muscles will be minimized. Lastly, understanding the anatomical variations of the PMi muscles is vital for several surgical procedures and clinical applications, including PMi release and nerve blocking [26].

## Conclusions

In conclusion, the systematic review has disclosed that, in humans, the PMi muscles have different anatomic patterns and heightened degrees of variations with regard to the origin and attachment/insertion points. The variations are attributable to the evolutionary history of humans along with their embryological developments. Despite the PMi insertion regularly occurring at the scapula’s coracoid process, the insertion has been observed to occur at the proximal humerus, the glenohumeral capsule, the supraspinatus tendon, and the coracoacromial ligament. Further, in humans, all the variations and anomalies, including the variations in the pectoralis minor insertion points, the pectoralis abdominis, and the deep fascicles have been observed to have considerable functional and clinical implications.

Therefore, the present study has contributed additional knowledge on human anatomy and evolution and has highlighted the different and important clinical issues in human medicine. The rare variations in the pectoralis minor have to be considered during surgical practices. For instance, the breast augmentation methods utilizing subglandular and submuscular planes in implant introduction are among the widely acknowledged and employed procedures. Following the performance of vasectomy, the breast implants are mainly placed in two distinct ways, including the under-muscle application that entails the placement of the implant between the pectoralis minor and pectoralis major muscles. Thus, the cognition of the pectoralis minor muscle variations is important for suck types of surgical practices. The study briefly emphasized the morphological variabilities of the pectoralis minor muscles. It is important that the infrequent variational attachments of the pectoralis minor muscles are considered during surgical practices. Further, the anatomical knowledge and understanding of the variations in pectoralis minor muscles and the pectoral region’s accessory muscles is vital to both surgeons and orthopedicians, particularly during the performance of shoulder arthroscopic procedures and chest wall surgeries. Also, the prior knowledge and comprehension of the pectoralis minor variations in relation to the pectoral region’s accessory muscles is prone to assist radiologists in conducting various diagnostic imaging techniques and procedures.
